# Enterocolitis Is a Risk Factor for Bowel Perforation in Neonates With Hirschsprung's Disease: A Retrospective Multicenter Study

**DOI:** 10.3389/fped.2022.807607

**Published:** 2022-02-07

**Authors:** Tianqi Zhu, Guofeng Zhang, Xinyao Meng, Jixin Yang, Yonghua Niu, Ying He, Heying Yang, Xiaofeng Xiong, Jiexiong Feng

**Affiliations:** ^1^Department of Pediatric Surgery, Tongji Medical College, Tongji Hospital, Huazhong University of Science and Technology, Wuhan, China; ^2^Hubei Clinical Center of Hirschsprung Disease and Allied Disorders, Wuhan, China; ^3^Department of Pediatric Surgery, The First Affiliated Hospital of Zhengzhou University, Zhengzhou, China; ^4^Department of Neonatal Surgery, Wuhan Children's Hospital, Wuhan, China

**Keywords:** Hirschsprung disease, bowel perforation, enterocolitis, risk factor, neonates

## Abstract

**Background and Aim:**

We evaluated the clinical features of neonatal Hirschsprung's disease (HD)-associated bowel perforation (perforated HD) and investigated risk factors related to it.

**Methods:**

We retrospectively collected clinical data of neonates (<1 month of age) with perforated HD from multicenters in China from January 2006 to December 2019. A total of 142 patients (6.7%) with perforated HD were enrolled in the study. A 1:2 matching method was used to compare the clinical information of HD patients with and without bowel perforation during the neonatal period. The risk factors for bowel perforation were identified using univariate and multivariate logistic risk regression analyses.

**Results:**

Perforation site was present in the proximal ganglionic bowel in 101 (71.1%) cases and the distal aganglionosis segment in 41 (28.9%) cases. Adjacent marginal tissue from the perforated intestine revealed varying degrees of inflammatory cell infiltration, and the severity of enterocolitis was higher in the proximal ganglionic bowel than in the distal aganglionosis segment (*p* < 0.05). In the univariable and multivariable logistic analyses, clinical symptoms, such as vomiting (adjusted OR = 2.06, 95% CI: 2.01–2.88, *p* < 0.05), and inflammation index in hematologic tests, such as neutrophil proportion (adjusted OR = 1.09, 95% CI: 1.05–1.33, *p* < 0.05) and CRP (adjusted OR = 2.13, 95% CI: 1.01–3.27, *p* < 0.05) were associated with increased risk for perforated HD.

**Conclusion:**

Clinical Hirschsprung disease-associated enterocolitis (HAEC) highly correlated with perforated HD. Timely treatment of HAEC should be appropriate therapeutic approaches to prevent perforated HD.

## Introduction

Hirschsprung's disease (HD) is a congenital intestinal malformation, characterized by the absence of ganglion cells in the submucosal and myenteric plexus of the bowel. Neonates with HD frequently present with clinical signs of abdominal distension, vomiting, constipation, and failure to pass meconium. HD is subdivided by the proximal extent of the aganglionic bowel segment into short-segment HD (rectosigmoid colon, SS-HD), long-segment HD (transverse or left colon, LS-HD), total colonic aganglionosis (TCA), and total intestine aganglionosis (TIA) (1). Bowel perforation (perforated HD), a serious complication of HD, mostly occurs during the neonatal period (2). According to a nationwide survey conducted in Japan from January 1, 2008, to December 31, 2012, perforated HD accounted for 1.8% of all neonatal perforations (3). Perforated HD is mostly associated with severe consequences, including high staged-operation rate, long parenteral feeding, and potentially worse prognosis.

Newman et al. first reviewed the perforation in neonates with HD and speculated that the mechanism of perforation could be related to increased intraluminal pressure (2). Afterward, Arliss et al. reported a neonatal appendiceal perforation diagnosed with SS-HD. Microscopic sections of the appendix demonstrated periappendicitis without transmural inflammation. Therefore, the authors suggested that both the special anatomical structure of the neonatal appendix and excessive luminal pressure could be responsible for appendiceal perforation (4). In addition, Yamamoto et al. reported a case of cecal perforation with SS-HD and suggested the cause of perforation to be ischemia, secondary to the vascular accident of the intestinal wall (5). It has been difficult to understand the true etiology of perforated HD until now due to the little systematic data related to epidemiologic findings or risk factors associated with perforated HD.

Hirschsprung disease-associated enterocolitis (HAEC) is one of the most common complications of HD, which can occur at any time from the neonatal period into adulthood. Teitelbaum et al. reported that neonates with HAEC had a mortality rate of 5% and a morbidity rate of 30% (6). Histological evidence of HAEC consists of a few features including crypt abscess, leukocyte aggregates, and ulceration in the affected intestinal wall (7). Significant clinical features associated with HAEC during the acute phase include bilious vomiting, fever, marked abdominal distension, and even bowel perforation (8). Therefore, we hypothesized that HAEC was the cause of perforation in neonates HD. The present work collected information on patients diagnosed with perforated HD from multicenters in China for over 10 years and evaluated the clinical features, aims to provide a comprehensive understanding of the risk factors associated with perforated HD.

## Methods

In this study, the medical records of neonates with HD treated between January 2006 and December 2019 at three different tertiary children's medical centers in China, were reviewed. The inclusion criteria were neonates ≤ 1-month-old with bowel perforation and diagnosed by HD. HD was diagnosed based on postoperative histological findings. Patients diagnosed with TIA, necrotizing enterocolitis (NEC), imperforate anus, intestinal atresia, and meconium plug syndrome were excluded. Neonates who were brought from outside the three participating hospitals were also excluded from the study.

In total, 2,119 consecutive patients with neonatal HD were admitted to our centers, of whom 142 (6.7%) were diagnosed with perforated HD. All clinical data of patients with perforated HD were recorded, including gestational age at birth, birth weight, gender, type of HD, site of perforation, site of stoma, and outcomes. Among them, there were 108 males (76.1%) and 34 females (23.9%), with a median age of 5 days at perforation (IQR: 2–18 days). All patients who developed bowel perforation were term or near-term infants, which was similar to that reported in a previous study (9). Histopathology of the adjacent marginal tissue of the perforated intestine from patients was retrospectively collected and reviewed by two pathologists independently.

A retrospective matched case–control study was conducted to assess the risk factors associated with perforated HD in neonates. For each index case suffering from neonatal HD with bowel perforation, we matched two controls, that is, neonatal HD without bowel perforation (1:2 matching method). The control population was matched for factors including age (days of bowel perforation in cases, ±3 days), gestational age, the same admissible hospital, and the same year of hospitalization.

Cases and control subjects were identified by reviewing their electronic medical records that included demographic, hospital course, and outcome data for all admissions to the hospitals. The potential risk factors for perforated HD included sex, *in vitro* fertilization, gestation details, type of delivery, family history, birth weight, delayed meconium defecation (>24 h after birth), abdominal distention or vomiting after birth, feeding timing (oral feeding or not), feeding pattern (breast or formula milk, or both), complicated with multi-disorders, blood transfusion (erythrocytes, thrombocytes, or fresh frozen plasma), and the latest laboratory blood examination. The risk factor data were collected for each case and its matched control subjects from birth to the day before bowel perforation in the case subject. For example, if the patient had developed bowel perforation on day 5, data about the hospital course in the case and assigned control subjects were collected from birth through day 4. Certain variables, such as birth Apgar score, blood pressure, and pH value, were incomplete and therefore excluded from analyses. This retrospective case–control study was approved by the medical ethics committee of the Tongji Hospital, Huazhong University of Science and Technology, Wuhan, China (No. 2019-S108). The trial was registered at Clinical Trial (No: NCT05044741). The study was performed according to the Declaration of Helsinki. All authors had access to the study data and reviewed and approved the final manuscript.

### Statistical Analysis

Continuous variables are reported as mean and standard deviation (SD), or median and interquartile range (IQR) according to normal and non-normal distribution, respectively. Categorical variables are expressed as frequencies and percentages. Various demographic and clinical characteristics, as well as established risk factors for bowel perforation, were compared between cases and control subjects using univariate logistic regression analyses. The results are presented as two-sided *p*-values, unadjusted odds ratios (ORs), and corresponding 95% confidence intervals (95% CIs). Patients' characteristics showing significant trends (*p* < 0.05) in association with important variables were evaluated through a multivariate conditional logistic regression analysis. Data are presented as two-sided *p*-values, adjusted OR (aOR), and corresponding 95% CI. All data were analyzed using Stata version 15. Significance in the adjusted and unadjusted analyses was established with *p* < 0.05.

## Results

**Figure 1:**
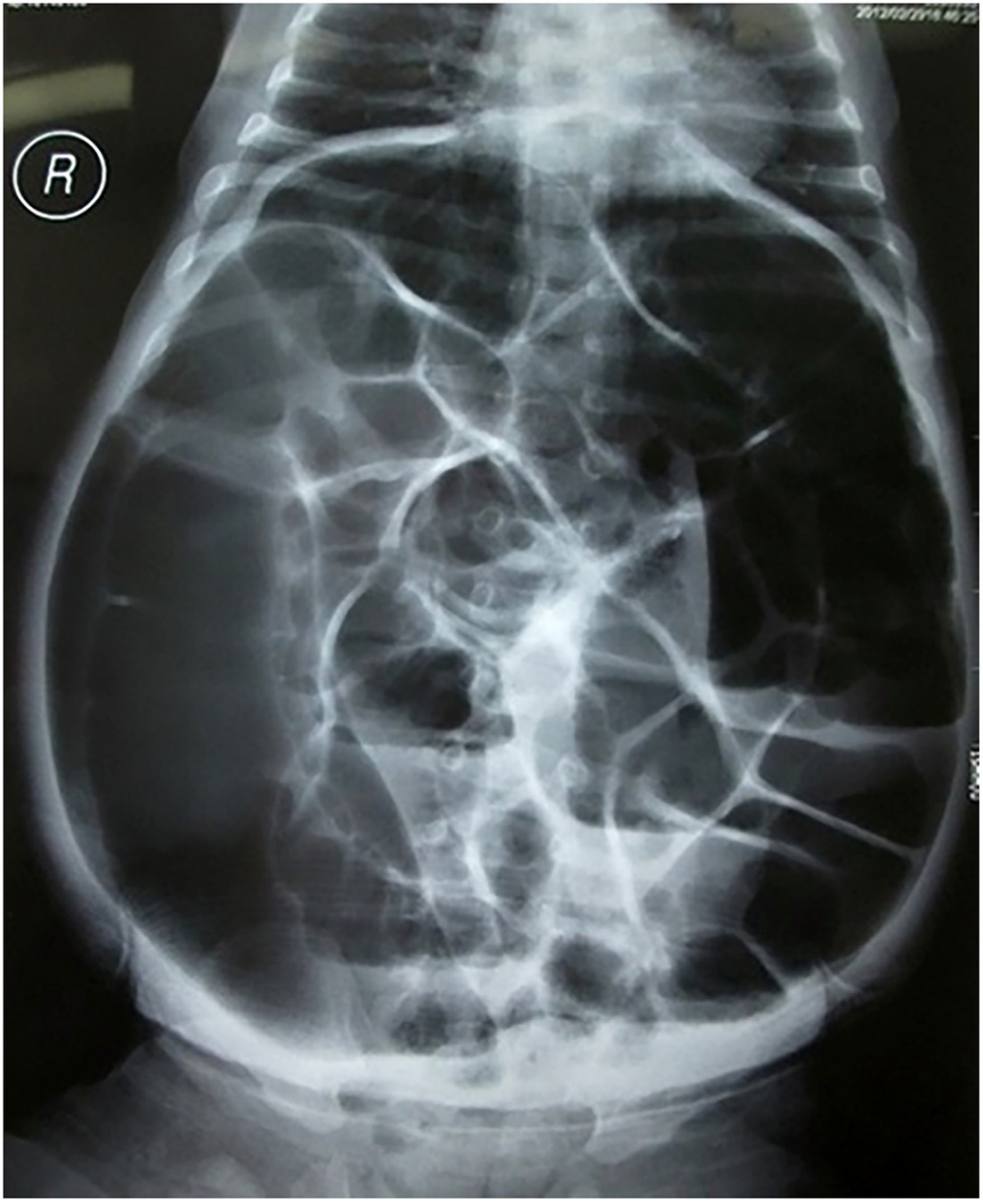
Typical anterior-posterior plain radiographs showed extensive intestinal and colon flatulence and multiple fluid levels.

The clinical characteristics of patients are summarized in Table 1. In patients with perforated HD, vomiting was reported in 108 cases (76.1%) and abdominal distension was reported in 129 cases (90.8%). In addition, 30 cases (21.1%) were hospitalized with intestinal obstruction as the major complaint, and 112 cases (78.9%) underwent bowel perforation during perinatal hospitalization. Typical orthostatism plain films showed extensive intestinal and colon flatulence and multiple fluid levels (Figure 1). With respect to the type of HD, 78 (54.9%) cases were diagnosed with SS-HD, 44 (31.0%) cases were diagnosed with LS-HD, and 20 (14.1%) cases were diagnosed with TCA (Table 2).

**Table 1:** Demographic and outcome data in neonates with perforated HD (*n* = 42).

**Characteristics**
Age (days), median (IQR)	5 (2–18)
Gestational age (weeks), median (IQR)	38.1 (37.4–40.1)
**Type of HSCR**
Short-segment	78 (54.9%)
Long-segment	44 (31.0%)
Total colonic aganglionosis	20 (14.1%)
**Site of Perforation**, ***n*** **(%)**
Sigmoid colon	60 (42.3%)
Descending colon	7 (4.9%)
Cecum	34 (23.9%)
Ileum	41 (28.9%)
**Site of stoma**, ***n*** **(%)**
Sigmoid colon	21 (14.8%)
Transverse colon	46 (32.4%)
Ileum	75 (52.8%)

**Table 2:** A comparison of risk factors for HD bowel perforation in neonates and control subjects.

**Variables**	**Perforated HSCR (*n* = 142)**	**Non-perforated HSCR (*n* = 284)**	**Unadjusted OR (95% CI)**	***P*-value**
Male gender, *n* (%)	108 (76.1%)	189 (66.5%)	1.63 (0.16–2.41)	0.49
Birth weight (kg), median (IQR)	3.1 (2.9–3.4)	3.5 (2.9–4.0)	0.35 (0.11–1.17)	0.09
Primigravid (*n*) (%)	81 (57.0)	176 (62.0%)	0.87 (0.43–5.04)	0.53
Primiparity (*n*) (%)	74 (52.1%)	155 (54.6%)	0.82 (0.35–4.19)	0.75
Cesarean delivery, *n* (%)	81 (57.0%)	148 (52.1%)	1.23 (0.24–2.78)	0.76
*In vitro* fertilization, *n* (%)	30 (21.1%)	80 (28.2%)	0.82 (0.87–3.71)	0.57
Complications/syndromes, *n* (%)	28 (19.7%)	76 (26.8%)	0.45 (0.07–2.76)	0.39
Transfusion history, *n* (%)	27 (19.0%)	40 (14.1%)	1.21 (0.14–3.14)	0.68
Family history, *n* (%)	7 (4.9%)	14 (4.9%)	NA	1
Meconium > 24 h, *n* (%)	128 (90.1%)	203 (71.4%)	1.46 (0.05–4.50)	0.13
Constipation after birth, *n* (%)	122 (85.9%)	256 (90.1%)	0.88 (0.24–10.60)	0.64
Fever (°C), median (IQR)	36.5 (5.8–7.4)	36.7 (6.0–7.8)	0.84 (0.32–3.61)	0.55
Abdominal distention, *n* (%)	129 (90.8%)	216 (76.1%)	1.34 (0.06–1.98)	0.23
Vomiting, *n* (%)	108 (76.1%)	102 (35.9%)	3.39 (2.65–4.77)	**<0.01**
Oral feeding, *n* (%)	115 (80.9%)	213 (75.0%)	1.25 (0.17–3.31)	0.71
**Type of feeding**, ***n*** **(%)**
Breast milk	47 (33.1%)	108 (38.1%)	0.86 (0.57–1.62)	0.89
Formula	34 (23.9%)	48 (16.9%)	1.43 (0.66–3.22)	0.10
Both	34 (23.9%)	60 (21.1%)	1.25 (0.87–2.56)	0.14
Did not feed	27 (19.1%)	68 (23.9%)	0.78 (0.66–3.04)	0.23
**Diagnosis**, ***n*** **(%)**
SS-HSCR	78 (54.9%)	176 (62.0%)	0.87 (0.41–1.82)	0.71
LS-HSCR	44 (31.0%)	27 (9.5%)	1.63 (0.78–3.56)	0.06
TCA	20 (14.1%)	81 (28.5%)	0.34 (0.23–1.35)	0.09
White blood cells count (10^9^/L), median (IQR)	11.3 (9.6–17.9)	10 (8.9–12.1)	1.13 (0.33–2.24)	0.16
Neutrophil proportion (%), mean (SD)	82.3 ± 12.3	54.8 ± 23.5	2.34 (1.24–6.23)	**0.03**
Neutrophil count (10^9^/L), mean (SD)	8.1 ± 4.5	6.7 ± 3.3	1.23 (0.73–3.24)	0.13
Red blood cell count (10^12^/L), median (IQR)	3.76 (3.24–8.21)	4.59 (3.72–7.21)	0.21 (0.09–5.12)	0.67
Hemoglobin (g/L), mean (SD)	112 ± 34	132 ± 24	0.43 (0.25–3.11)	0.44
Platelet count (10^9^/L), mean (SD)	207 ± 87	255 ± 101	0.67 (0.32–4.21)	0.32
Albumin levels (g/L), mean (SD)	43 ±12	37 ± 11	2.41 (0.55–5.23)	0.65
C-reactive protein (mg/L), median (IQR)	14.8 (6.6–20.5)	2.5 (1.7–3.5)	3.65 (1.06–4.57)	**0.02**

*Note: Values in bold are significant*.

**Figure 2:**
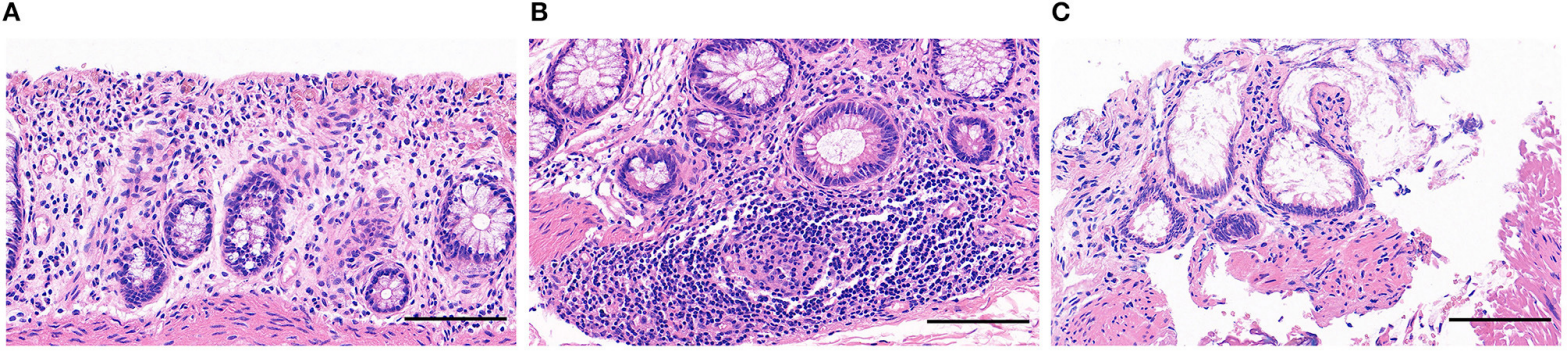
Pathological manifestation of adjacent marginal tissue from the perforated intestine. **(A)** Mild inflammatory cell infiltration, with focal mucosal ischemic necrosis. (Grade I); **(B)** HAEC Grade II: Infiltration of a large number of inflammatory cells with crypt dilation and mucin retention. (Grade II); **(C)** Infiltration of a large number of inflammatory cells with crypt abscesses and epithelium destruction. (Grade III).

The site of perforation bowel was sigmoid colon in 60 (42.3%) cases, descending colon in 7 (4.9%) cases, cecum in 14 (9.9%) cases, appendix in 20 (14.1%) cases, and terminal ileum in 41 (28.9%) cases. Of these, perforation site in 101 (71.1%) cases was present in the proximal ganglionic bowel, including 70 cases with SS-HD and 31 cases with LS-HD. Perforation site in 41 (28.9%) cases was present in the distal aganglionosis segment, including 8 cases with SS-HD, 13 case with LS-HD, and 20 cases with TCA. In cases with SS-HD or LS-HD, the majority of the perforation sites (89.7 and 70.5%, respectively) were located in the proximal ganglionic bowel. However, the perforation site was located in the cecum or appendix in all TCA cases. Adjacent marginal tissue from the perforated intestine was obtained in 122 (85.9%) patients, histological examination of the adjacent marginal tissue from the perforated intestine revealed mild inflammatory cell infiltration [Grade I according to HAEC histological grading system (7)] in 37 samples (30.3% of tested specimens); severe inflammatory cell infiltration, with significant signs of variable crypt dilation (Grade II) in 31 samples (25.4% of tested specimens); and numerous crypt abscesses with the destruction of the epithelium (Grade III) in 54 samples (44.3% of tested specimens) (Figure 2). Concretely, in Grade II and Grade III samples, the severe inflammatory response was more common in the proximal ganglionic bowel (61 cases, 71.8% of Grade II and Grade III) than in the distal aganglionosis segment (24 cases, 28.2% of Grade II and Grade III) (*p* < 0.05).

All cases received emergency surgical enterostomy, including sigmoid colostomy in 21 cases (14.8%), transverse colostomy in 46 cases (32.4%), and ileostomy in 75 cases (52.8%). During the diversion of luminal flow, HAEC occurred in 7 cases (4.9%), and constipation after enterostomy occurred in 9 cases (6.3%), which were resolved by daily bowel irrigation until radical colectomy. Staged stoma closure and pull-through colectomy were performed 3–6 months later. Seven cases (4.9%) were lost to follow-up. The remaining patients survived through follow-ups with no need for re-operation after radical colectomy. No deaths were reported.

Univariable logistic analysis revealed no significant differences with respect to sex, birth weight, primigravid, primiparity, delivery type, *in vitro* fertilization, complicated with multi-disorders, blood transfusion, family history, fever, abdominal dilation after birth, feeding practice, and HD type. Furthermore, no significant difference was observed between the groups for red blood cell (RBC) count, hemoglobin, platelet count, and albumin levels before diagnosis with perforation. The complicated with multi-disorders/syndromes in the perforation group included congenital heart disease (*n* = 10), inguinal hernia (*n* = 5), polydactyly (*n* = 4), Down's syndrome (*n* = 3), Waardenburg-Shah syndrome (*n* = 2), hypoxic-ischemic encephalopathy (*n* = 1), and ectopic pancreas (*n* = 3). Seventy-six complicated with multi-disorders/syndromes were reported in the control group: congenital heart disease (*n* = 25), inguinal hernia (*n* = 14), polydactyly (*n* = 14), syndactylia (*n* = 8), Down's syndrome (*n* = 5), Meckel's diverticulum (*n* = 5), hypoxic-ischemic encephalopathy (*n* = 3), and congenital hydrocephalus (*n* = 2).

On the day of birth, patients with perforated HD reported a higher rate of vomiting compared with the non-perforation groups (76.1 and 35.9%, respectively). In addition, they reported a significantly high risk of perforated HD (unadjusted OR = 3.39, 95% CI: 2.65–4.77, *p* < 0.01). The latest laboratory data before diagnosis of perforated HD revealed significantly increased neutrophil proportion (unadjusted OR = 2.34, 95% CI: 1.24–6.23, *p* < 0.05) and C-reaction protein (CRP) (unadjusted OR = 3.65, 95% CI: 1.06–4.57, *p* < 0.05) in the infants with perforation (Table 2).

Multivariable analysis revealed that vomiting (adjusted OR = 2.06, 95% CI: 2.01–2.88, *p* < 0.05), increased neutrophil proportion (adjusted OR = 1.09, 95% CI: 1.05–1.33, *p* < 0.05), and CRP (adjusted OR = 2.13, 95% CI: 1.01–3.27, *p* < 0.05) were associated with an increased risk of perforated HD (Table 3).

**Table 3:** Multivariate regression analysis of risk factors for HD bowel perforation in neonates.

**Variables**	***P*-value**	**Adjusted OR**	**(95% CI)**
Vomiting	0.04	2.06	2.01–2.88
Neutrophil percentage (%), mean (SD)	0.02	1.09	1.05–1.33
C-reactive protein (mg/L), median (IQR)	0.02	2.13	1.01–3.27

## Discussion

Bowel perforation is an uncommon and severe complication of HD. The rate of perforation among patients with HD has been reported to range from 3 to 6%, with variations among different centers (2, 10, 11). Most of the studies over the past decades are based on cases reports, and little information is available regarding the risk factors for bowel perforation in HD. It is essential to identify neonates HD at high risk of developing bowel perforation and provide prompt intervention.

Although associated risk factors contributing to the development of perforated HD have been considered, most of the previous studies have directly related it to increased luminal pressure caused by distal bowel obstruction (2, 11). However, certain questions remained unanswered, e.g., compared with other common neonatal obstructive diseases, such as imperforate anus (most predisposed to intestinal perforation due to high luminal pressure) (9), the high-risk period for perforation was normally between 36 and 48 h after birth (12). This is considerably earlier than that for HD perforation. Furthermore, spontaneous perforation of the imperforate anus was estimated to occur in 2% of neonates (13), which is also lower than the occurrence of HD perforation.

To elucidate the mechanism of perforated HD, we reviewed the adjacent marginal tissue from perforated intestinal of 122 (85.9%) patients. Histopathological analysis revealed varying degrees of infiltration of inflammatory cells in all samples. Furthermore, the inflammatory response was more severe in the proximal ganglionic colon than in the distal aganglionosis colon. HAEC is the most serious and potentially life-threatening complication of HD. To further understand the pathogenesis of HAEC, we have previously reported the activation of different types of inflammatory cells, especially pro-inflammatory macrophages (M1), resulting in crypt abscesses and mucosal damage, and that HAEC occurs preferentially in the proximal dilated colon of HD patients (14). This finding was consistent with the pathological results described above. The majority of the perforation occurred in the proximal ganglionic bowel, which could be ascribed to the occurrence of severe enterocolitis. Due to the limited literature reporting associated pathological HAEC with perforated HD (15), our study was the first to study inflammatory changes in the intestinal tissue in perforated HD.

To further support the hypothesis that HAEC was associated with the occurrence of perforated HD, our logistic analysis confirmed that inflammatory biomarkers in the laboratory tests of blood, including CRP, and neutrophil proportion, were significantly related to increased rates of perforated HD. Indeed, these biomarkers reflected the serum inflammatory status in these cases (16). Therefore, clinical HAEC is associated with high risks of perforated HD, in line with local and systemic inflammation.

In contrast, our regression results also showed vomiting as an independent risk factor for perforated HD (aOR: 2.06, CI: 2.01–2.88), indicating that increased luminal pressure may also contributed to gastrointestinal tract perforation. Surprisingly, certain dietary factors, including feeding pattern and feeding time, were not associated with perforated HD. As another index of increased intraperitoneal pressure, abdominal distention was insignificantly related to regression in the present study, which could be attributed to the prevalence of abdominal distention in neonates with perforated or non-perforated HD (90.8 and 76.1%, respectively) (17). However, we still lack specific evidence about excessive luminal pressure as an independent cause of perforated HD. In this regard, a prospective study to further detect dynamic changes in the intraperitoneal pressure would be beneficial.

In addition, Arliss et al. (4) have reported appendicular perforation in a 7-day-old boy diagnosed with SS-HD. Histological evidence provided by the authors showed peri-appendicitis without transmural inflammation. Therefore, authors related the special funnel-shaped structure of the neonatal appendix opening into the cecum to the potential point of maximum tension, resulting in pressure perforation in the presence of distal obstruction (18). This speculation supplements the explanation of the phenomenon of perforation at the cecum and appendix (9.9 and 14.1% in all cases, respectively), where the most common perforation sites of TCA were located in the current study.

Given that HAEC is proved as a risk factor of perforated HD, we need to diagnose HAEC promptly and choose the best treatment strategy. When there are obvious symptoms of neonatal ileus with delay in the passage of meconium, but mechanical ileus such as imperforate anus is excluded, neonatal HD should be considered (19). The hematologic tests are routinely monitored, once the inflammatory index, such as CRP value, elevated neutrophil proportion, and white blood cells account, are unusually increased, with feeding difficulties, vomiting, lethargy, with or without fever, diagnosis of HAEC should be made and symptomatic treatment aimed at HAEC should be timely provided (20). Broad-spectrum antibiotic therapy and intestinal decompression are recommended as critical approaches in the initial management of confirmed HAEC, with additional management strategies, including fluid resuscitation and correction of electrolyte disturbances (8). Timely intestinal decompression is essential to control intestinal inflammation because distal bowel obstruction is also a strong causative factor for the HAEC episode (7). Noticeably, for fulminant neonate HAEC, especially in LS-HD or TCA, rectal washouts have a high risk of iatrogenic perforation (21). In case of failure to adequately decompress the bowel or perforation, an emergency operation with the diversion of luminal flow is required. Because perforation may still occur in the aganglionic or transitional segment, where stoma may result in persistent obstruction, the transition zone must be carefully detected to determine the appropriate location of the stoma. An intraoperative frozen section is used to ensure that the stoma is located in the ganglionic bowel, and a multipoint biopsy should be performed (15).

### Limitations

Because this study was the first to explore the causes of bowel perforation in HD, it had certain limitations. First, HD patients were recruited in the study over a decade, during which treatment strategies must have changed drastically. Second, the sample size was limited and underpowered to detect slight changes between the groups. However, we limited the effect of these factors by matching HD patients and controls through a multicenter study design approach. Third, some data were not available for all patients due to the retrospective nature of the study, indicating that certain risk factors associated with perforation remained undetected. Further prospective larger-sample studies that can contribute more details would provide a multivariable model with a better fit.

## Conclusion

In conclusion, perforated HD is more likely to occur in full-term or near-term neonates. The perforation site is more likely to be located in the proximal ganglionic bowel. Combined with a severe pathological inflammatory response, especially in the proximal dilated bowel, and independent risk factors, including high CRP value and elevated neutrophil proportion in hematologic tests, HAEC is proved to be associated with perforated HD. Timely treatment of HAEC with or without the diversion of luminal flow would be the appropriate approach to prevent perforated HD.

## Data Availability Statement

The raw data supporting the conclusions of this article will be made available by the authors, without undue reservation.

## Ethics Statement

The studies involving human participants were reviewed and approved by the Medical Ethics Committee of the Tongji Hospital, Huazhong University of Science and Technology, Wuhan, China (No. 2019-S108). Written informed consent to participate in this study was provided by the participants' legal guardian/next of kin. Written informed consent was obtained from the minor(s)' legal guardian/next of kin for the publication of any potentially identifiable images or data included in this article.

## Author Contributions

JF designed the research study. TZ, GZ, HY, XX, and JF performed the research. TZ and XM analyzed data. TZ, JY, YN, and YH wrote the initial draft of the manuscript. HY, XX, and JF provided critical assessments during the revision process leading to the final submitted manuscript. All authors have reviewed and approved the final version of this manuscript.

## Funding

This study was supported by Hubei Provincial Key Research and Development Program (No. 2020BCB008) and Clinical Research Pilot Project of Tongji Hospital (No. 2019YBKY026).

## Conflict of Interest

The authors declare that the research was conducted in the absence of any commercial or financial relationships that could be construed as a potential conflict of interest.

## Publisher's Note

All claims expressed in this article are solely those of the authors and do not necessarily represent those of their affiliated organizations, or those of the publisher, the editors and the reviewers. Any product that may be evaluated in this article, or claim that may be made by its manufacturer, is not guaranteed or endorsed by the publisher.
